# Neuroprotective effect of the hairy root extract of *Angelica gigas* NAKAI on transient focal cerebral ischemia in rats through the regulation of angiogenesis

**DOI:** 10.1186/s12906-015-0589-4

**Published:** 2015-04-01

**Authors:** Tae Woo Oh, Ki-Ho Park, Hyo Won Jung, Yong-Ki Park

**Affiliations:** Department of Herbology, College of Korean Medicine, Dongguk University, Gyeongju, 780-714 South Korea; Korean Medicine R&D Center, Dongguk University, Gyeongju, 780-714 Republic of Korea

**Keywords:** *Angelica gigas*, Korean angelica, Focal cerebral ischemia, Angiogenesis, Blood-brain barrier, Neuroprotection

## Abstract

**Background:**

In this study, we investigated the neuroprotective effect of the hairy root extract of *Angelica gigas* NAKAI (Angelica Gigantis Radix) on transient focal cerebral ischemia in rats through the regulation of angiogenesis molecules.

**Methods:**

Male Sprague-Dawley rats were induced focal cerebral ischemia by a transient middle cerebral artery occlusion (tMCAO) for 90 min, and then orally administrated with the water extract of *A. gigas* hairy roots (AG). After 24 h reperfusion, infarction volume and the changes of BBB permeability were measured by TTC and Evans Blue (EB) staining. The neuronal cell damage and the activation of glial cells were assessed by immunohistochemistry in the ischemic brain. The expression of angiogenesis-induced proteins such as angiopoietin-1 (Ang-1), and vascular endothelial growth factor (VEGF), inflammatory protein such as intercellular adhesion molecule-1 (CAM-1), tight junction proteins such as ZO-1, and Occludin and the phosphorylation of phosphatidylinositol 3-kinase (PI3K)/AKT were determined in the ischemic brains by Western blot, respectively.

**Results:**

The treatment of AG extract significantly decreased the volumes of brain infarction, and edema in MACO-induced ischemic rats. AG extract decreased the increase of BBB permeability, and neuronal death and inhibited the activation of astrocytes and microglia in ischemic brains. AG extract also significantly increased the expression of Ang-1, Tie-2, VEGF, ZO-1 and Occludin through activation of the PI3K/Akt pathway. AG extract significantly increased the expression of ICAM-1 in ischemic brains.

**Conclusions:**

Our results indicate that the hairy root of AG has a neuroprotective effect in ischemic stroke.

## Background

Brain stroke is an important cause of extensive health problems throughout the world. Stroke causes a localized process of ischemic cell death in the brain, but triggers a regenerative response in brain tissue adjacent to the ischemic area of cell death [1-4]. Re-establishment of functional microvasculature through angiogenesis promotes stroke recovery [5]. During cerebral angiogenesis, the initial vascular plexus forms mature vessels by sprouting, branching, pruning and differential growth of endothelial cells, and recruitment of supporting cells such as pericytes and smooth muscle cells [6]. Angiogenesis and vascular maturation/remodeling are regulated by vascular endothelial growth factor (VEGF), angiopoietin (Ang)-1 and Ang-2, and the receptor tyrosine kinases, Tie-1 and -2 [7-10]. Cerebral endothelial cells perform essential functions including maintenance of the blood brain barrier (BBB) and regulation of vascular tone by release of vasoactive factors. Generally, the permeability of the blood vessels is caused after cerebral ischemia within 1-2 hours, and will increase with time, which is maintained up to 24 hours. Cerebral injury affects both of the BBB and autoregulation because the extent of flow during reperfusion and is these are correlated with neurological injury. Cerebral inflammation can eventually disrupt the BBB further by more extensive activation of resident cells like astrocytes and microglia, and infiltration of inflammatory cells, macrophages and leukocytes. As brain injury triggers the inflammatory response and aggravates the injury, the decreased expression of TNF-α and ICAM-1 indicate a diminished progression of injury. Inflammatory cytokine such as TNF-α will stimulates the expression of ICAM-1, leading to leukocyte adhesion and extravasation. Recent experimental studies show that systemic inflammation exacerbates neutrophil infiltration in the brain, altering the kinetics of the BBB tight junction disruption after experimental stroke in mice [11]. A transformation from transient to sustained BBB disruption caused by enhanced neutrophil-derived neurovascular MMP-9 is a critical mechanism underlying the exacerbation of ischemic brain injury by systemic inflammation, mediated through conversion of a transient to a sustained disruption of the tight junction protein [12].

VEGF is a pleiotropic angiogenic growth factor that is crucial in neovascular remodeling in the ischemic stroke. VEGF promotes angiogenesis, protects ischemic neurons from injury, has potent anti-inflammatory actions, and promotes brain plasticity, in addition to enhancing the recruitment and proliferation of neuronal precursors [13]. Angiopoietins (Ang-1 and Ang-2) are ligands for the endothelial-specific receptor tyrosine kinase, Tie-2 [14]. Acute alternation of VEGF and Ang-1 in the ischemic core may mediate BBB leakage, whereas up-regulation of VEGF/VEGF receptors and Ang/Tie-2 at the boundary zone may regulate angiogenesis with neovascularization in ischemic brain.

Ang-1 and VEGF in combination induce a synergistic angiogenic effect, and promote the formation of mature neovessels without the side effects on BBB permeability. Therefore, stroke promotes vascular stabilization and decreases BBB leakage, by increasing Ang1/Tie2 and VEGF/Flk1 expression, and both together promote angiogenesis and vascular maturation after stroke.

The root of *Angelica gigas* NAKAI (Umbelliferae; Angelica Gigantis Radix), known as Korean angelica, *A. gigas* (AG) root is a herbal medicine for the treatment of various circulatory disorders with female afflictions such as dysmenorrhea, amenorrhea, menopause, abdominal pain, migraine and arthritis [15]. AG has biological activities such as anti-cancer [15-18], anti-platelet aggregation [16], neuroprotection [17], anti-inflammatory, anti-oxidant [18] and anti-osteoclastogenesis [19] with several coumarin derivates including decursin decursinol, decursinol angelate, nodakenin, nodakenetin and umbelliferone [20,21]. In Oriental medicine, the root of AG is able to divide two parts, root body and hairy root according to their efficacy on tonify blood and promote blood circulation. For example, the body root has been used for blood deficiency syndrome, and the hairy root has been used for blood stasis. However, the effect of AG extract on the BBB permeability and angiogenesis with vascular stabilization has not been investigated. Stroke is an inflammatory disease caused by the extravasation of blood in the brain. Therefore, in this study, we evaluate the effect of the hairy root of AG on blood stasis and inflammation in ischemic brain through improving the blood disability. For this, we investigated the expression of angiogenesis-induced proteins, such as VEGF and Ang-1/Tie-2, and tight junction molecules such as Occludin and ZO-1 with the BBB permeability in transient middle artery cerebral occlusion (tMCAO)-induced ischemic stroke in rats, and investigated its action mechanism on the PI3K/Akt signaling pathway.

## Methods

### Preparation of AG extract

*A. gigas* (AG) roots were purchased from a medicinal materials company (Kwangmyungdang Medicinal Herbs, Ulsan, Republic of Korea) and authenticated by Y. K. Park, a botanist in the Department of Herbology, College of Oriental Medicine, Dongguk University (DUCOM), Republic of Korea. AG extract was prepared by the following procedure. The roots were boiled in distilled water for 3 h, filtered through a two-layer mesh and Whatman No. 1 paper, and concentrated under vacuum. The final yield of concentrated extract was 29.1% of the dried powder. AG extract was stored at 4°C, and dissolved in saline prior to use.

### Animals

Male Sprague-Dawley (SD) rats weighing an average of 280 ± 10 g (Orient Bio Inc., Gyeonggi-do, Rep. of Korea) were used in the experiments. The animals were housed under controlled environmental conditions at an ambient temperature of 23 ± 1°C, relative humidity of 50 ± 10% and 12 h light/dark cycle with free access to food and water. All animals were handled according to the animal welfare guidelines issued by the Korean National Institute of Health and the Korean Academy of Medical Sciences for the care and use of laboratory animals and approved by the Institutional Animal Care and Use Committee of Dongguk University.

### Preparation of ischemic stroke rat model

The ischemic stroke rat model was prepared by transient middle cerebral artery occlusion (tMCAO) and reperfusion following a standard procedure [22]. Rats were anesthetized with 4% isoflurane and maintained using 1% isoflurane in a mixture of 30% oxygen and 70% nitrous oxide, during the surgical procedure. Rectal temperature was measured with a rectal probe and was kept at 37°C using a heating pad (FHC Inc., ME, USA). The left common carotid artery (CCA) was exposed and separated carefully from the vagus nerve and ligated at the more proximal side through a right paramedian incision. The external carotid artery (ECA) was ligated. The occipital artery and the pterygopalatine artery were coagulated. Ischemia was produced by advancing the tip of a rounded 3-0 nylon suture into the ICA through the ECA. After placement, the intraluminal suture was secured with suture tied around the ECA. Reperfusion was produced by withdrawal of the intraluminal suture. In sham group, the ECA was surgically prepared for the insertion of the filament, but the filament was not inserted.

All animals were randomly divided into six groups (n = 18 per a group): group I, sham-operation (Sham); group II, tMCAO/reperfusion-induced ischemic group with saline treatment (vehicle); group III, vehicle with AG-treated group at dose of 10 mg/kg; group IV, vehicle with AG-treated group at dose of 25 mg/kg; group V, vehicle with AG-treated group at dose of 50 mg/kg; and group VI, vehicle with AG-treated group at dose of 100 mg/kg. AG extract was administrated orally once 90 min after tMCAO, and then all animals were reperfused for 24 hr. All animals were euthanized by decapitation after 24 h of reperfusion. The brain tissues were harvested for next experiments, and were used for the measurement of brain infarction (n = 6 per a group), edema (n = 3 per a group), morphological changes of neuronal cells (n = 3 per group), Western blot (n = 3 per group), and BBB permeability (n = 3 per group).

### Measurement of infarct volume

All animals were euthanized by decapitation after 24 h of reperfusion. The brain tissues were harvested and cut into 2-mm coronal slices starting 2mm from the frontal pole. Each slice was stained with 2,3,5,-triphenyltetrazolium chloride (TTC) for measurement of the infarction volumes. In TTC stain, the infarction was observed in the unstained part, whereas the normal part was stained red. The infarction volume was calculated as the infarct volume (mm^3^) per brain by a computerized imaging analyzing system (Adobe Systems Incorporated, San Jose, CA). Therefore, infarction volumes were expressed as a percentage of the contralateral hemisphere volume using the formula: (the area of the intact contralateral hemisphere—the area of the intact region of the ipsilateral hemisphere) to compensate for edema formation in the ipsilateral hemisphere.

### Measurement of the water content in brain

After 24 h of reperfusion, all animals were killed and brains were collected. The pons and olfactory bulb were removed and the brain wet weight (ww) measured. All brains were dried at 110°C for 24 h, and the brain dry weight (dw) measured. Whole water content in brains was calculated using following formula: (ww-dw)/ww × 100 as an index for brain edema [23].

### Nissl staining

The brain sections were de-paraffinized in xylene, sequentially rehydrated in graded ethanol and then immersed in 0.01 M PBS (pH 7.4). The sections were microwaved for 5 min in 0.01 M sodium citrate buffer (pH 6.0), cooled to room temperature, and then washed three times for 3 min in PBS. The sections were incubated in 3% hydrogen peroxide for 20 min to eliminate endogenous peroxidase activity, and then washed in PBS. The sections were stained with 0.2% thionine treated with Nissl stain for histological assessment of ischemic damage. The number of neuronal cells in the border of the infarct area was counted.

### Immunohistochemistry

Twenty-four h after reperfusion, anaesthetized rats were perfused with 100 mL of 4% paraformaldehyde in 0.1 M phosphate buffer (pH 7.4). Brains were removed rapidly and post-fixed for 24 h in the same fixative. The paraffin-embedded brain tissues were cut on a cryo-ultramicrotome (Leica, Wetzlar, Germany) into serial 10-μm coronal sections. For histological assessment of ischemic damage, paraffin-embedded brain sections were stained with hematoxylin-eosin (H&E) staining. The brain sections were deparaffinized and non-specific endogenous peroxidase activity blocked with 3.0% H_2_O_2_ for 5-min at room temperature (RT). After washing with PBS, the sections were reacted with rat anti-neuronal nuclei (NeuN) mAb (1:100, Millipore, Bedford, MA, USA) as a neuronal marker, rat anti-glial fibrillary acidic protein (GFAP) mAb (1:50, Abcam, Cambridge, MA, USA) as an astrocyte marker, and mouse anti-rat CD11b mAb (1:50, BD Pharmingen, San Diego, CA, USA) as a microglia marker for 24 h at 4°C, then incubated with biotinylated anti-mouse, -goat, and -rabbit immunoglobulins for 30 min at RT. After again washing with PBS, the sections were incubated with streptoavidin-conjugated horseradish peroxidase (HRP) for 30 min at RT. Finally, the sections were reacted with a solution containing diaminobenzidine (DAB) and hydrogen peroxide (0.001%). The sections were counterstained with toluidine blue or hematoxylin, dehydrated and embedded with Permount. Histopathological changes of ischemic brains were observed under microscope with 400× magnification.

### Western blot

Twenty-four h after reperfusion, brain tissues were collected from all animals, homogenized with a RIPA buffer [50 mM Tris–HCl (pH 7.4), 150 mM NaCl, 1mM PMSF, 1mM EDTA, 1% Triton X-100, 0.5% sodium deoxycholate, and 0.1% SDS)] for the isolation of protein. Protein samples were electrophoresed on 10% gradient sodium dodecyl sulfate (SDS)-polyacrylamide gel (Bio-Rad, Hercules, CA, USA) and electro-transferred to nitrocellulose (NC) membranes. The membranes were incubated with blocking buffer (5% skimmed milk in 25mm Tris-HCl, pH 8.0, 125mm NaCl, 0.1% Tween 20) for 1 h at RT, followed by incubation with primary antibodies for anti-Ang-1 mAb (1:500 Santa Cruz Biotechnology, Santa Cruz, CA, USA), and anti-Tie-2 mAb (1:500 Santa Cruz Biotechnology), anti-VEGF mAb (1:1000 Santa Cruz Biotechnology), anti-ICAM-1 mAb (1:500, Sigma, St Louis, MO, USA), anti-ZO-1 mAb (1:500, Invitrogen, Carlsbad, CA, USA), anti-Occludin mAb (1:500, Abcam, Cambridge, MA, USA), and anti-β-actin mAb (1:2000; Sigma) overnight at 4°C. The membranes were washed with blocking buffer without milk, and then incubated with horseradish peroxidase-conjugated secondary antibody. Immunoreactive proteins were detected by the enhanced chemiluminescence system (ECL, Sigma) and serial exposures were made on X-ray film (Hyperfilm ECL, Amersham International). The target proteins were analyzed and quantified by a computer-associated densitometry.

### Evans Blue staining

Evans blue (EB, 2%) as a BBB permeability tracer was injected intravenously 4 h in femoral vein before euthanasia. The brains were removed and coronal sections from bregma-1 to 1 mm were divided into the right and left hemispheres. The sections were homogenized with 50% trichloroacetic acid and then centrifuged at 15,000 rpm for 20 min. The intensity of EB was determined by a spectrophotometer at 620 nm (excitation) and 680 nm (emission). Calculations were based on the external standards dissolved in the same solvent, and the amount of EB extravasation was quantified as micrograms per ischemic hemisphere [24,25].

### Statistical analysis

All data are represented as means ± standard deviation (SD), and statistical analysis was performed by GraphPad program 5.0 software. The significance level of each group was performed by the Duncan's Multiple Comparison Test after Kruskal-Wallis non-parametric ANOVA. Probability level less than 0.05 was considered as statistically significant from vehicle.

## Results

### Effect of AG extract on brain infarction

**Figure 1:**
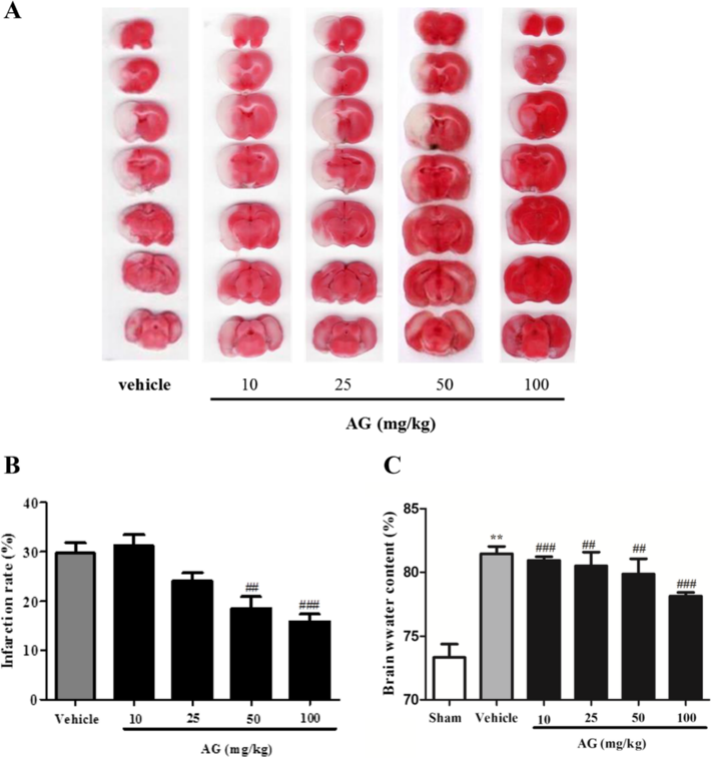
**Effects of AG extract on the brain infarction in MCAO-induced ischemic rats.** After MCAO for 90 min and reperfusion for 24 h, brain tissues were coronary sectioned (2 mm thick) and then stained with TTC. **(A)** Representative photographs of TTC staining of coronal brain sections. **(B)** The histogram of brain infarction volumes (n = 6 per a group). **(C)** The histogram of brain edema (n = 3 per a group). Values are expressed as mean ± SD of each group. ^**^P < 0.01 vs. sham, and ^##^P < 0.01, and ^###^P < 0.01 vs. vehicle.

To evaluate the neuroprotective effect of AG extract on ischemic damages, we measured the infarction volumes in MCAO-induced ischemic rats by TTC staining. MCAO in rats was induced brain infarction in the vehicle group (29.7 ± 5.2%). AG extract at doses of 10, 25, 50 and 100 mg/kg decreased the infarction volumes with 31.1 ± 5.6%, 24.0 ± 3.9%, 18.5 ± 5.8%, and 15.8 ± 3.7%, respectively (Figure 1A, B). AG extract at 50 and 100 mg/kg significantly decreased the infarction volume (P < 0.01 and P < 0.001, respectievly) compared with that of vehicle group.

Next, we measured the water content in the ipsilateral hemisphere of brains in MCAO-induced ischemic rats by wet-dry method. The brain water content significantly increased (P < 0.01) in the vehicle group with 81.7 ± 1.2%, compared with the saline-treated sham group (Figure 1C). AG extract at doses of 25, 50, and 100 mg/kg significantly decreased water content by 80.4 ± 2.2%, 79.8 ± 3.2%, and 78.1% ± 0.9%, respectively, compared with that of the vehicle group.

### Effect of AG extract on ischemic damages in neuronal cells

**Figure 2:**
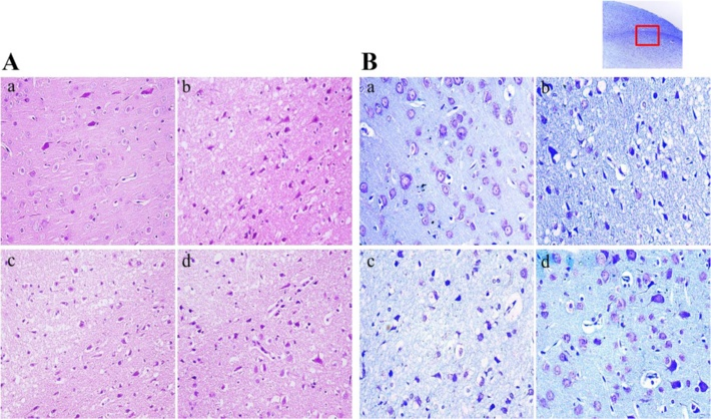
**Effect of AG extract on neuronal damage in MCAO-induced ischemic rats.** After MCAO/reperfusion, brain tissues were stained by H&E **(A)** and Nissl **(B)**. The morphological changes of neuronal cells in a boundary between cortex and penumbra (square box) were observed by a microscope (original magnification ×400). a, sham group; b, vehicle: MCAO-induced ischemic group; c, AG extract at 50 mg/kg-treated group in vehicle; and d, AG extract at 100 mg/kg-treated group in vehicle. The photograph is a representative image of three different tissues.

To evaluate the neuroprotective effect of AG extract on ischemic neuronal damages, we investigated the morphological changes of neuronal cells in the ischemic hemisphere of MCAO-induced ischemic rats. In the saline-treated sham group, neuronal cells were observed to be normally intact and well-arranged morphologically with abundant cytoplasm and clear nucleus in the cortex of rats (Figure 2A). MCAO in rats induced morphological changes in neuronal cells with marked shrinking, vacuolation, eosinophilic cytoplasm and triangulated pyknotic nuclei in the ischemic penumbra of rats. AG extract at doses of 50 and 100 mg/kg inhibited the morphological changes of neuronal cells.

We also investigated neuronal apoptosis in the cortex of MCAO-induced ischemic rats by Nissl staining. In the saline-treated sham group, most of the neuronal cells were Nissl-negative normal morphology (Figure 2B). After MCAO in rats, the numbers of Nissl-stained apoptotic neuronal cells with aberrant morphology increased in the vehicle group. AG extract at doses of 50 and 100 mg/kg inhibited neuronal apoptosis similar to the sham group.

### Effect of AG extract on the activation of astrocytes and microglia in ischemic brain

**Figure 3:**
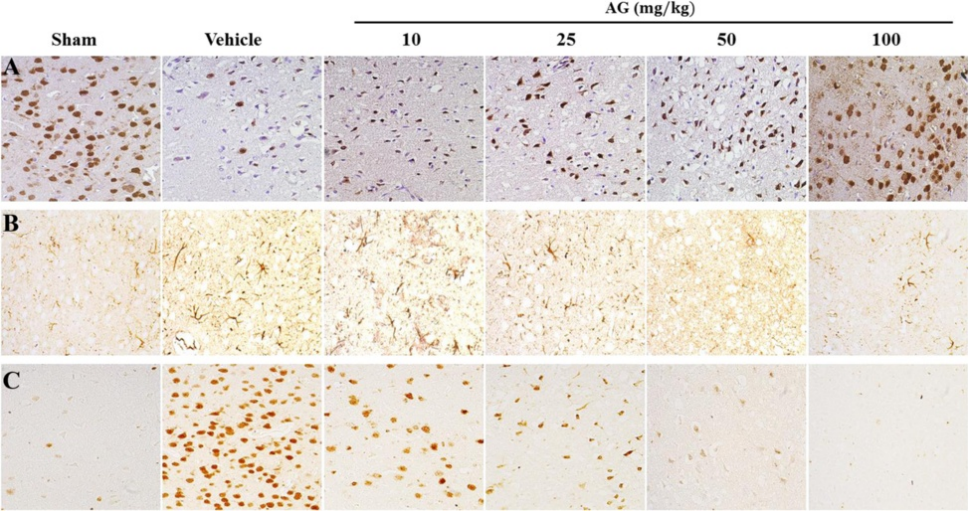
**Effects of AG extract on the activation of glial cells in the brain of MCAO-induced ischemic rats.** After MCAO/reperfusion, brain tissues were immune-stained by anti-NeuN **(A)**, anti-GFAP **(B)** and anti-OX-42 **(C)** antibodies. The morphological changes of neuronal cells **(A)**, astrocytes **(B)**, and microglia **(C)** were observed in the penumbra of ischemic rats by microscope (original magnification ×400). The photograph is a representative image of three different tissues.

To investigate the effect of AG extract on the activation of inflammatory cells in ischemic brain, we observed the morphological changes of astrocytes and microglia, and neuronal death by immunohistochemistry. MCAO demarcated an infarction range from vital areas and induced loss of NeuN-positive neuronal cells (Figure 3A) and activation of inflammatory cells such as GFAP-positive astrocytes (Figure 3B), and OX-42-positive microglia (Figure 3C) in the ischemic penumbra. AG extract at doses of 25, 50 and 100 mg/kg in MCAO-induced ischemic rats increased NeuN-neuronal cells, while decreasing inflammatory cells such as GFAP-positive astrocytes and OX-42-positive microglia in a dose-dependent manner. These inflammatory cells were maintained at resting morphology by treatment with AG extract at 200 mg/kg similar to the sham group.

### Effect of AG extract on the expression of angiogenesis-regulated molecules in ischemic brain

Ang-1 and Tie-2 receptor tyrosine kinase have wide-ranging effects on angiogenesis, inflammation and vascular extravasation [26]. Tie-2 activation by Ang-1 stimulation may down-regulate inflammatory responses in angiogenesis, and up-regulate the expression of adhesion molecules such as ICAM-1, VCAM-1 and E-selectin on brain endothelial cells during inflammation. Therefore, we investigated the expression of Ang-1, Tie-2, VEGF, ICAM-1 and tight junction molecules such as ZO-1 and Occludin in ischemic brains of MCAO rats.

**Figure 4:**
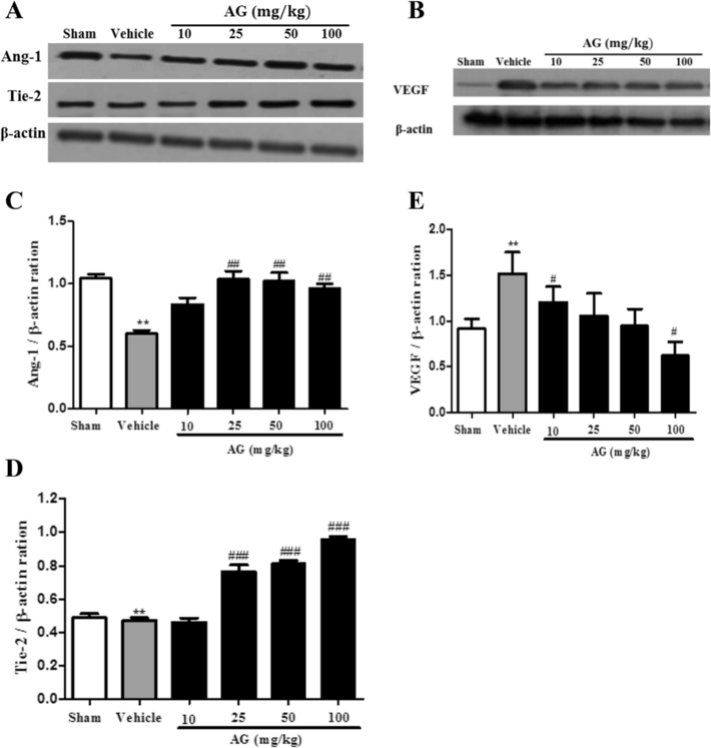
**Effect of AG extract on the expression of angiogenesis-regulated molecules in the brain of MCAO-induced ischemic rats.** After MCAO/reperfusion, the protein was isolated from brain tissues, and detected the expression of Ang-1, Tie-2 **(A)** and VEGF **(B)** by western blot. β-actin was used as a control. Relative folds of Ang-1 **(C)**, Tie2 **(D)** and VEGF **(E)** were calculated by normalization to β-actin. Data in the histogram are expressed as means ± SD of three independent experiments (n = 3 per group). ^**^P < 0.01 vs. sham; and ^#^P < 0.05, ^##^P < 0.01, and ^###^P < 0.001 vs. vehicle.

**Figure 5:**
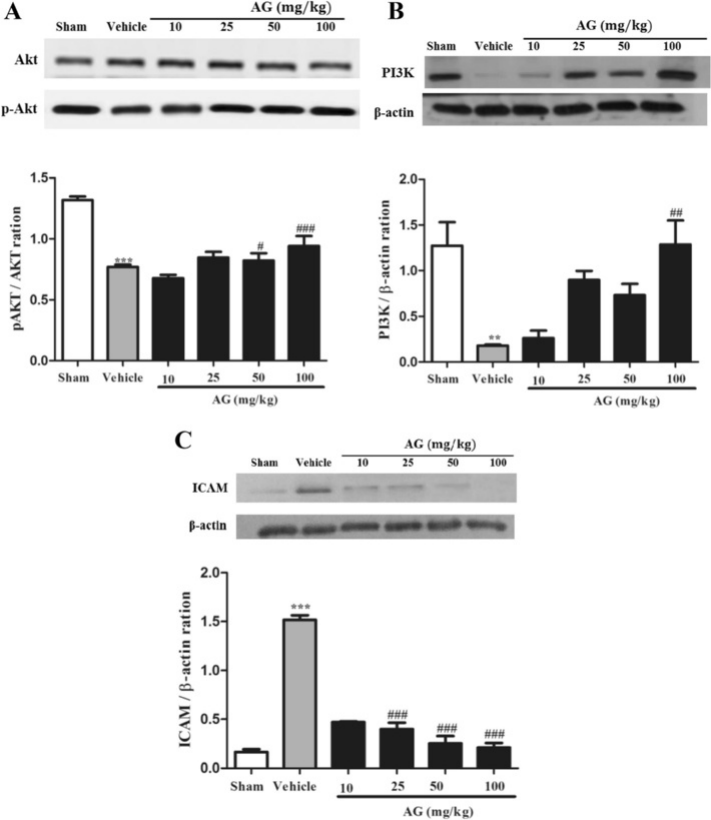
**Effect of AG extract on the expression of Akt, PI3K and ICAM in the brain of MCAO-induced ischemic rats.** After MCAO/reperfusion, the protein was isolated from brain tissues, and assayed for expression of phosphorylated- or whole forms of Akt **(A)**, PI3K **(B)** and ICAM **(C)** by western blot. β-actin was used as a control. Relative folds of p-Akt and PI3K and ICAM were calculated by normalization to Akt and β-actin, respectively. Data in the histogram are expressed as means ± SD of three independent experiments (n = 3 per group). ^**^P < 0.01, and ^***^P < 0.001 vs. sham; and ^#^P < 0.05, ^##^P < 0.01, and ^###^P < 0.001 vs. vehicle.

As shown in Figure 4, the expression of Ang-1, Tie-2 and VEGF was significantly decreased in MCAO-induced ischemic brains compared with sham group (P < 0.01, respectively). The treatment of AG extract at doses of 25, 50 and 100 mg/kg in MCAO rats significantly increased the expression of Ang-1, and Tie-2 (Figure 4A,C,D) and decreased the expression of VEGF (Figure 4B,E) compared with the vehicle group. In addition, AG extract at dose of 100 mg/kg significantly induced phosphorylation of Akt (Figure 5A), and expression of PI3K (Figure 5B). ICAM-1 expression was strongly inhibited by AG extract treatment at all doses in MCAO rats (Figure 5C).

**Figure 6:**
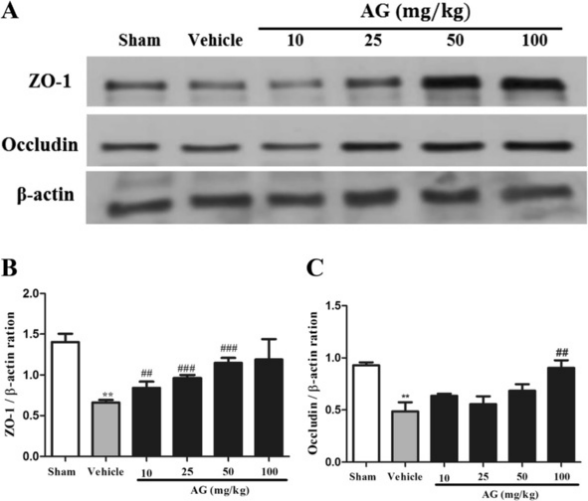
**Effect of AG extract on the expression of ZO-1 and Occludin in the brain of MCAO-induced ischemic rats.** The expression of ZO-1 and Occludin was detected in MCAO-induced ischemic brain by western blot **(A)**. β-actin was used as a control. Relative folds of ZO-1 **(B)** and Occludin **(C)** were calculated by normalization to β-actin. Data in the histogram are expressed as means ± SD of three independent experiments (n = 3 per group). ^**^P < 0.01 vs. sham; and ^##^P < 0.01, and ^###^P < 0.001 vs. vehicle.

As shown in Figure 6A, the expression of tight junction molecules such as ZO-1 and Occludin was significantly decreased in ischemic brains compared with sham group (P < 0.01 and P < 0.01, respectively). The treatment of AG extract in MCAO rats increased the expression of ZO-1 (Figure 6B) and Occludin (Figure 6C) in a dose-dependent manner.

### Effect of AG extract on the BBB Leakage

**Figure 7:**
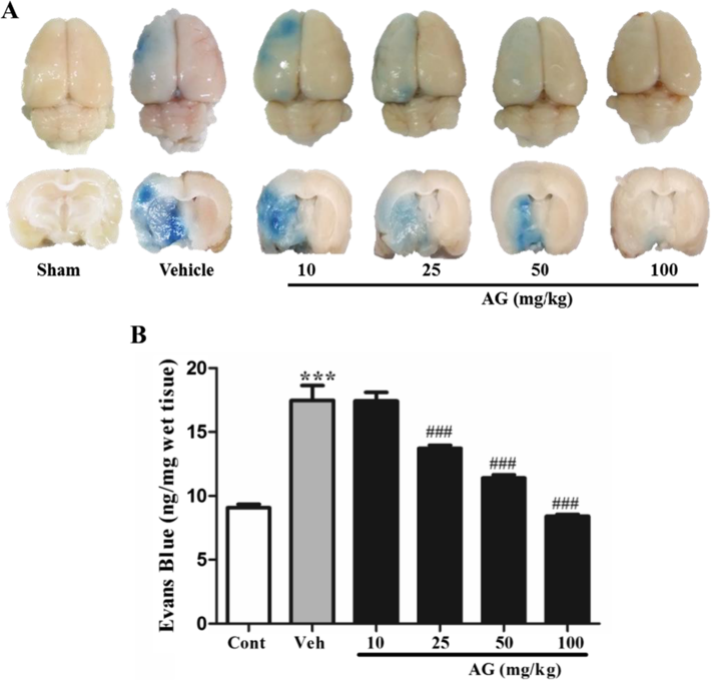
**Effect of AG extract on the changes of water content in the brain of MCAO-induced ischemic rats.** After MCAO/reperfusion, Evans blue was refused into the brains through i.p. injection. The brains were isolated, and measured the blue-positive area **(A)** by photo and the blue density in brain extract by spectrophotometry **(B)**. Data in the histogram are expressed as means ± SD of three independent experiments (n = 3 per group). ^***^P < 0.001 vs. sham; and ^###^P < 0.001 vs. vehicle.

To investigate the effect of AG extract on the BBB damage in ischemic brain, we measured BBB permeability by Evans blue staining. As shown in Figure 7, Evans blue (EB) stain indicates BBB leakage in MCAO-induced ischemic rats. AG extract at doses of 25, 50 and 100 mg/kg significantly reduced BBB leakage (P < 0.001, respectively) compared with that of the vehicle group. AG extract at dose of 100 mg/kg strongly inhibited, by 48%, the BBB disruption.

## Discussion

Cerebral ischemia induces a complex cascade of biochemical and molecular changes [27]. In this study, we investigated the neuroprotective effect of AG water extract in MCAO-induced ischemic rats and the working mechanism linked in the BBB destruction. Our results show that the treatment with AG extract in MCAO rats effectively reduced the brain infarction by inhibiting the activation of glial cells such as astrocytes and microglia that are crucial in neuroinflammation of cerebral ischemia [25]. Astrocytes and microglia are potent regulators of brain capillary endothelial cell function and profoundly influence the morphogenetic events underlying the organization of the vessel wall [28,29]. Therefore, our results indicate that AG extract has a neuroprotective effect in ischemic conditions like a cerebral stroke by regulating the glial cell activation.

VEGF is a key regulator of vasculogenesis and embryogenic angiogenesis. In the central nervous system (CNS), VEGF is essential in wound healing for vascular endothelial proliferation and survival, and in the proliferation of astrocytes and the maintenance during the repair of brain injury [30,31]. The upregulation of VEGF is reported in neurons, astrocytes, microglia, and blood vessels of animal models of stroke including MCAO [32-34]. Recently, the expression of Ang-1 and Ang-2 is also known in focal cerebral ischemia [35-37], and their cell type-specific expression is closely related with glioblastoma angiogenesis [38]. In particular, Ang-1 protects the adult vasculature against plasma leakage [39], and has been found to have a strong anti-inflammatory effect in angiogenesis, through the activation of Tie-2 receptor [40]. Ang-1may also be considered a switch that controls the transition from the inflammatory in vascular endothelial cells [41]. Signaling transduction by the Tie-2 is activated by cell survival pathway such as PI3K/Akt, leading to vascular stabilization [42]. Angiogenic regulators including VEGF/Ang-1 do not stimulate the growth of endothelial cells, but regulate their survival through the PI3K/Akt pathway [43]. Following cerebral ischemia, these angiogenic regulators could participate in survival and repair in the ischemic brain.

Unlike VEGF, Ang-1 and -2, their receptor Tie-2, and the associated receptor Tie-1 exert their functions at larger stages of vascular development e.g. during vascular remodeling and maturation with blood vessel formation, depending on the availability of VEGF. In this study, the treatment of AG extract in MCAO rats increased the expression of VEGF, Ang1 and Tie-2 in ischemic brain. In addition, AG extract promotes the expression of tight junction molecules such as ZO-1 and occluding in ischemic brain. Hyper-stimulation both of VEGF and Ang1 expression in mouse brain increases microvessel density with the maintenance of ZO-1 protein expression [26], and the combination of submaximal doses of Ang1 and VEGF enhances blood vessel formation in ischemic condition [44,45]. Our result indicates that AG extract induces angiogenesis after ischemic damage in the brain by increasing Ang-1and Tie-2 expression.

The BBB is a highly complex structure, separating the extracellular fluid of the CNS from the blood of CNS vessels. A wide range of neurological conditions, including stroke, epilepsy, Alzheimer’s disease, and brain tumors, is associated with dysfunction of the BBB [46,47]. In addition, BBB impairment is involved in secondary inflammation and neuronal damage, thus contributing to disease pathogenesis. In this study, treatment with AG extract reduced the BBB destruction after MCAO/reperfusion in rats.

Tight junction is an effective barrier between the endothelial cells in the BBB [48,49]. Several tight junction-associated molecules such as Claudins, occludin, junctional adhesion molecule, accessory proteins, and cytoskeletal proteins (actin etc.) interact to maintain the tight junctions [48-50]. Therefore, tight junction proteins are subject to changes in expression, subcellular localization, post-translational modification and protein–protein interactions under both physiological and pathophysiological conditions [50]. Focal cerebral ischemia induces BBB disruption, and the loss of BBB integrity allows intravascular proteins and fluid to penetrate into the cerebral parenchymal extracellular space, thereby incurring vasogenic edema formation and further brain damage [51,52]. In this study, the expression of ZO-1 and Occludin protein was increased in ischemic brain by AG extract treatment in a dose-dependent manner. These results indicate that AG extract can effectively prevent neuronal damage from BBB leakage in ischemic rats.

## Conclusion

In this study, the hairy root extract of AG inhibits the brain infarction, edema, and BBB leakage in MCAO-induced ischemic rats through the inhibition of glial activation, and the increase of Ang-1, Tie-2, VEGF and tight junction proteins, ZO-1 and Occludin and the activation of PI3K/Akt. Our results indicate that the hairy root of AG has a neuroprotective effect in ischemic stroke.
